# Innate Immune Functions of Astrocytes are Dependent Upon Tumor Necrosis Factor-Alpha

**DOI:** 10.1038/s41598-020-63766-2

**Published:** 2020-04-27

**Authors:** Kyla R. Rodgers, Yufan Lin, Thomas J. Langan, Yoichiro Iwakura, Richard C. Chou

**Affiliations:** 1 Department of Medicine, Geisel School of Medicine at Dartmouth, Dartmouth College, One Medical Center Drive, Lebanon, NH 03756 USA; 2 Departments of Neurology, Pediatrics, and Physiology and Biophysics, Jacobs School of Medicine and Biomedical Sciences, State University of New York at Buffalo, Buffalo, NY 14203 USA; 3 Hunter James Kelly Research Institute, New York State Center of Excellence Bioinformatics & Life Sciences, Buffalo, NY 14203 USA; 4 Research Institute for Biomedical Sciences, Tokyo University of Science, 2669 Yamazaki, Chiba, 278-0022 Japan

**Keywords:** Innate immunity, Cellular neuroscience

## Abstract

Acute inflammation is a key feature of innate immunity that initiates clearance and repair in infected or damaged tissues. Alternatively, chronic inflammation is implicated in numerous disease processes. The contribution of neuroinflammation to the pathogenesis of neurological conditions, including infection, traumatic brain injury, and neurodegenerative diseases, has become increasingly evident. Potential drivers of such neuroinflammation include toll-like receptors (TLRs). TLRs confer a wide array of functions on different cell types in the central nervous system (CNS). Importantly, how TLR activation affects astrocyte functioning is unclear. In the present study, we examined the role of TLR2/4 signaling on various astrocyte functions (i.e., proliferation, pro-inflammatory mediator production, regulatory mechanisms, etc) by stimulating astrocytes with potent exogenous TLR2/4 agonist, bacterial lipopolysaccharide (LPS). Newborn astrocytes were derived from WT, *Tnfα*^*−/−*^, *Il1α*^*−/−*^*/Il1β*^*−/−*^, and *Tlr2*^*−/−*^*/Tlr4*^*−/−*^ mice as well as Sprague Dawley rats for all *in vitro* studies. LPS activated mRNA expression of different pro-inflammatory cytokines and chemokines in time- and concentration-dependent manners, and upregulated the proliferation of astrocytes based on increased ^3^H-thymidine update. Following LPS-mediated TLR2/4 activation, TNF-α and IL-1β self-regulated and modulated the expression of pro-inflammatory cytokines and chemokines. Polyclonal antibodies against TNF-α suppressed TLR2/4-mediated upregulation of astrocyte proliferation, supporting an autocrine/paracrine role of TNF-α on astrocyte proliferation. Astrocytes perform classical innate immune functions, which contradict the current paradigm that microglia are the main immune effector cells of the CNS. TNF-α plays a pivotal role in the LPS-upregulated astrocyte activation and proliferation, supporting their critical roles in in CNS pathogenesis.

## Introduction

Astrocytes are a specialized subtype of glia present throughout the central nervous system (CNS)^1^. These star-shaped cells were initially described by German pathologist Rudolf Virchow as the “connective tissue of the brain”. As a result of this dismissive analogy, astrocytes were long regarded as functionally insignificant^2^. However, recent studies have revealed that astrocytes actually play diverse and critical roles in both CNS homeostasis and pathophysiology, many of which are currently being uncovered. Importantly, under normal circumstances, astrocytes are quiescent; however, in the presence of pathological stimuli, astrocytes re-enter the cell cycle, undergo morphological and biochemical changes, and produce pro-inflammatory cytokines^3,4^. The molecular mechanisms underlying these changes remain unknown.

One potential mechanism underlying this transition is the activation of toll-like receptors (TLRs). TLRs are a class of membrane-bound receptors called pattern recognition receptors (PRRs) that trigger innate immune responses. Innate immunity is a highly conserved system that is found in all animals and present from birth. This system is nonspecific and fast-acting, recognizing broad, evolutionarily conserved molecular patterns that trigger the same response upon each encounter rather than orchestrating a tailored response to each unique pathogen. Specifically, these evolutionarily conserved molecular patterns are molecules expressed by groups of pathogens (i.e., pathogen-associated molecular patterns [PAMPs]) or classes of molecules released by damaged tissues (i.e., damage-associated molecular patterns [DAMPs]). Generally, plasma membrane-bound TLRs recognize bacteria- (e.g., bacterial lipopolysaccharides [LPS]) or fungus-associated PAMPs (e.g., chitin), while endosomal TLRs recognize foreign nucleic acids (e.g., viruses)^5^. To date, 13 TLRs have been identified, ten of which are expressed in humans, including TLRs 2 and 4^5,6^. TLR stimulation triggers intracellular cascades that ultimately upregulate transcription of pro-inflammatory cytokines, interferons, or chemokines^6^.

In the CNS, TLRs are expressed in various cell types, including neurons, microglia, oligodendrocytes, and astrocytes^7–11^. In these cells, TLR signaling is linked to a wide array of non-immune functions such as neuronal differentiation^7^, cell fate determination (e.g., astrocytes vs. neurons)^9^, and neurite outgrowth^11,12^. In oligodendrocyte precursor cells, TLR signaling promotes apoptosis^8^. TLR2 and TLR4 in the CNS also generate particular interest as they can be activated by non-infectious internal ligands such beta-amyloid protein^13,14^. In microglia, activation of TLR2 and TLR4 by various ligands including beta-amyloid protein promotes classical innate immune responses^13,14^. Interestingly, both TLR2 and TLR4 are expressed on astrocytes^15^; however, the effects of these TLRs on astrocyte activation and effector functions are unclear. Given the vast diversity of TLR2/TLR4 functions on different CNS cells, including CNS immune cells microglia, and the significance of TLR2/TLR4 in the pathogenesis of both infectious and non-infectious conditions, understanding the roles of astrocytic TLR2/TLR4 is imperative.

In the present study, we examined the relationship between astrocytic TLR2/4 stimulation and astrocyte activation and functioning using a potent exogenous TLR ligand, LPS. We also explored possible regulatory mechanisms underlying the effects of TLR2/4 activation on reactive astrocytes.

## Results

### Bacterial LPS upregulates astrocytic production of pro-inflammatory cytokines and chemokines

Because TLRs differentially affect various CNS cell types, we first examined how TLR2/4 activation by one of the most potent exogenous ligands of TLR2/4, LPS, affected murine astrocytes. To test this, cultured primary astrocytes from newborn WT murine pups were purified with a Miltenyi magnetic bead isolation system (>99% purity, Supplemental Fig. 1) and then subcultured in 12-well plates at a density of 1 × 10^4^ cells/well. The cells were then treated with graded concentrations of LPS (0.1–10 ng/mL) or vehicle control (PBS, pH 7.4) for various lengths of time (0–6 h). At the end of each time point, total RNA was harvested from the cells, followed by qPCR analysis for mRNA expression of different pro-inflammatory mediators.

**Figure 1:**
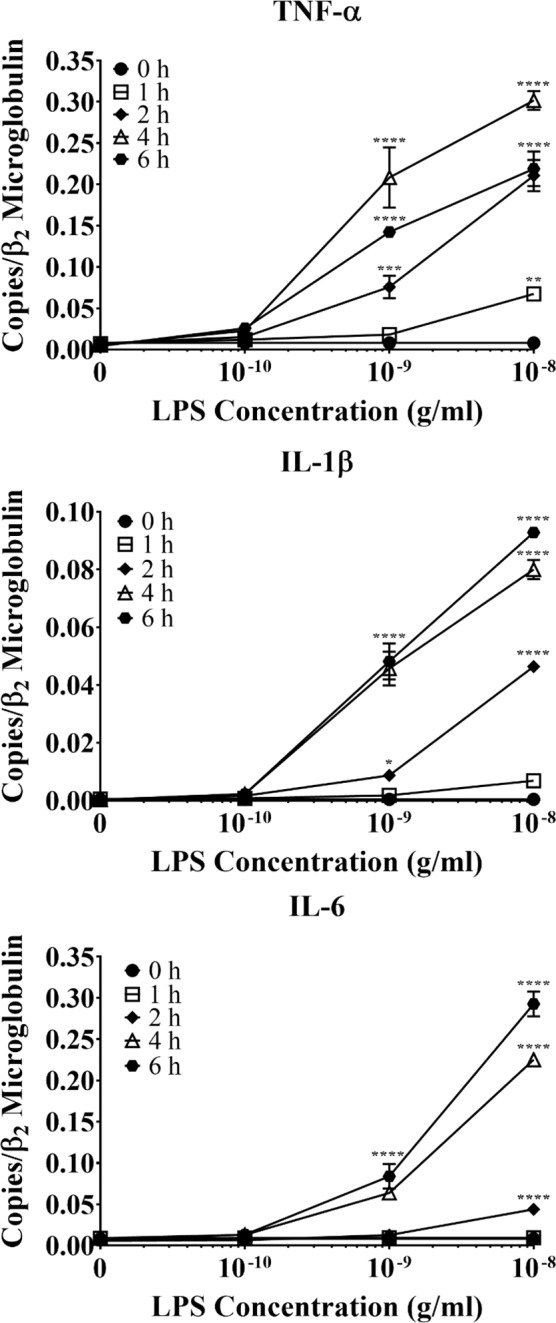
The effect of LPS on primary WT astrocyte production of pro-inflammatory cytokines. Purified and subcultured murine WT astrocytes were incubated with graded concentrations of freshly prepared bacterial LPS or vehicle control (PBS). Cultures were terminated at 0, 1, 2, 4, or 6 h after stimulation. Total RNA was then extracted, and mRNA expression of pro-inflammatory cytokines was determined by qPCR. *p < 0.05; **p < 0.01; ***p < 0.001; ****p < 0.0001 (n = 3, results show one representative experiment out of 4).

This revealed that LPS significantly increased astrocyte mRNA expression of various pro-inflammatory cytokines, i.e. TNF-α, IL-1β, and IL-6, in time- and concentration-dependent manners (Fig. 1). Specifically, the lowest concentration of LPS (0.1 ng/mL) did not upregulate any of the pro-inflammatory cytokines, regardless of the stimulation duration. However, 1 ng/mL LPS significantly upregulated mRNA expression of TNF-α and IL-1β following two (*p* < 0.001 and 0.05, respectively), four (*p* < 0.0001), and six hours (*p* < 0.0001) of stimulation, respectively. It also significantly upregulated IL-6 mRNA production after four and six hours (*p* < 0.0001). Alternatively, the highest concentration of LPS tested (10 ng/mL) significantly upregulated TNF-α mRNA expression following one (*p* < 0.01), two, four, and six hours of stimulation (*p* < 0.0001), while it significantly upregulated those of IL-1β and IL-6 after two, four, and six hours. These findings clearly demonstrate a temporal sequence in the LPS-induced astrocytic expression of the three pro-inflammatory cytokines: TNF-α → IL-1β → IL-6.

**Figure 2:**
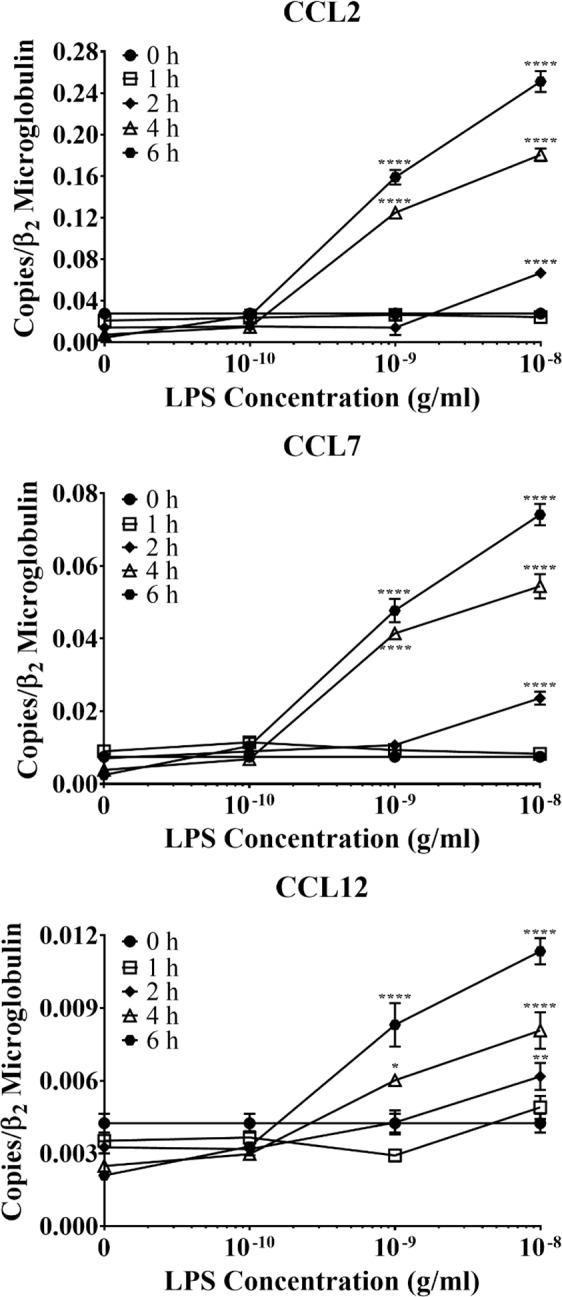
The effect of LPS on primary WT astrocyte production of CCR2 ligands. Purified and subcultured murine WT astrocytes were incubated with graded concentrations of freshly prepared bacterial LPS or vehicle control (PBS). Cultures were terminated at 0, 1, 2, 4, or 6 h after stimulation. Total RNA was then extracted, and mRNA expression of CCR2 ligands was determined by qPCR. *p < 0.05; **p < 0.01; ***p < 0.001; ****p < 0.0001 (n = 3, results show one representative experiment out of 4).

We next examined how LPS activation affected the ability of astrocytes recruit other immune cells, as recruitment and activation of resident microglia and circulating immune cells are key steps in the pathogenesis of several CNS diseases^16,17^. We were particularly interested in the expression of ligands for CCR2, a chemokine receptor that modulates the recruitment of resident microglia and peripheral monocytes in a mouse model of Alzheimer-like disease^16^. Our data revealed that LPS significantly upregulated the mRNA expression of three CCR2 ligands, i.e. CCL2, CCL7, and CCL12, in time- and concentration-dependent manners (Fig. 2). As with the pro-inflammatory cytokine expression, the lowest concentration of LPS (0.1 ng/mL) did not affect the production of any of the CCR2 ligands, regardless of stimulation duration. However, 1 ng/mL LPS upregulated mRNA production of all three CCR2 ligands following four or six hours of stimulation. Additionally, 10 ng/mL LPS significantly upregulated mRNA expression of all three chemokines following two, four, and six hours of stimulation; however, one hour of LPS stimulation did not upregulate the mRNA expression of any of the CCR2 ligands at any of the concentrations tested. Therefore, the mRNA expression of these three chemokines resembled the kinetics of IL-1β and IL6 in LPS-stimulated astrocytes, with delayed expression relative to TNF-α.

Overall, astrocyte CCL2 mRNA expression was most affected by LPS stimulation, as indicated by the production of CCL2 mRNA (0.25 copies/β_2_ microglobulin) after six hours of 10 ng/mL LPS stimulation. Such production was over 3 times higher than that of CCL7 (0.07 copies/β_2_ microglobulin) and approximately 25 times higher than that of CCL12 (0.01 copies/β_2_ microglobulin) during the same time period. It is unclear if astrocyte-derived CCL2, among CCL2, CCL7, and CCL12, plays a major role in the recruitment of resident microglia and peripheral monocytes following inflammatory stimulation by LPS without further recruitment essays.

**Figure 3:**
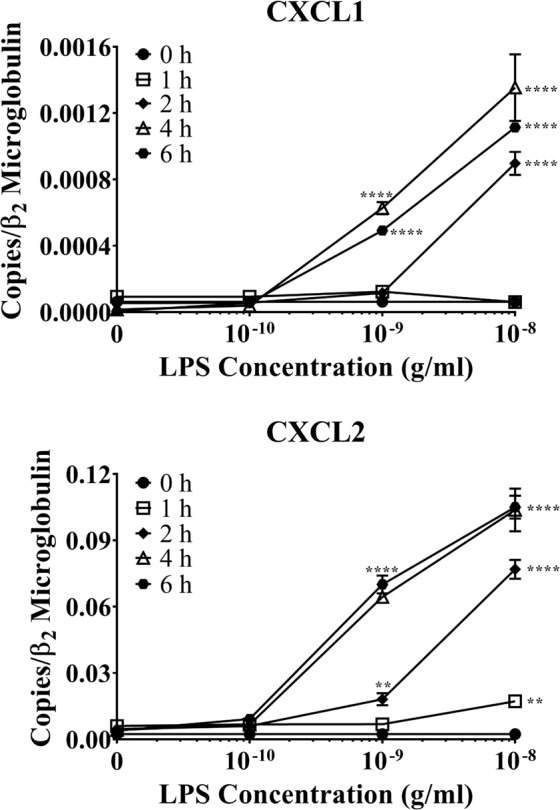
The effect of LPS on primary WT astrocyte production of neutrophil-active chemokines. Purified and subcultured murine WT astrocytes were incubated with graded concentrations of freshly prepared bacterial LPS or vehicle control (PBS). Cultures were terminated at 0, 1, 2, 4, or 6 h after stimulation. Total RNA was then extracted, and mRNA expression of neutrophil-active chemokines was determined by qPCR. *p < 0.05; **p < 0.01; ***p < 0.001; ****p < 0.0001 (n = 3, results show one representative experiment out of 4).

Because polymorphonuclear neutrophils are the predominant immune cells during acute inflammation, we next evaluated how TLR2/4 activation on astrocytes affected production of neutrophil-active chemokines. The chemokine IL-8, which acts through the CXCR2 receptor on neutrophils, is involved in the pathogenesis of several neuroinflammatory conditions^18–22^. While IL-8 is not expressed in mice, its murine functional equivalents, CXCL1 and CXCL2, act through CXCR2 on neutrophils. Therefore, we also examined the production of CXCL1 and CXCL2 mRNAs in LPS-stimulated murine astrocytes. LPS stimulation significantly increased mRNA expression of both chemokines in time- and concentration-dependent manners (Fig. 3). Consistent with our earlier findings regarding the production of pro-inflammatory cytokines and CCR2 ligands (Figs. 1 and 2), the lowest concentration of LPS did not affect the expression of either CXCL1 or CXCL2 mRNA, regardless of the stimulation duration. However, 1 ng/mL LPS did significantly upregulate CXCL1 mRNA expression following four and six hours of stimulation (*p* < 0.0001) and that of CXCL2 following two (*p* < 0.01), four, and six hours (*p* < 0.0001). Alternatively, two hours of 1 ng/mL LPS stimulation significantly upregulated CXCL2 (*p* < 0.01), but not CXCL1 mRNA. Finally, both neutrophil chemokines were significantly upregulated following two, four, and six hours of stimulation with 10 ng/mL (*p* < 0.0001). Therefore, our data suggest that CXCL2 might play a more important role than CXCL1 in neutrophil recruitment, as the amount of CXCL2 mRNA expressed by LPS-activated astrocytes was about 100 times higher than that of CXCL1, although recruitment assays have not been conducted to confirm such assertion. Therefore, future recruitment essays will further clarify the functional significance our findings.

### Astrocytic pro-inflammatory cytokines act in a self-regulatory manner

**Figure 4:**
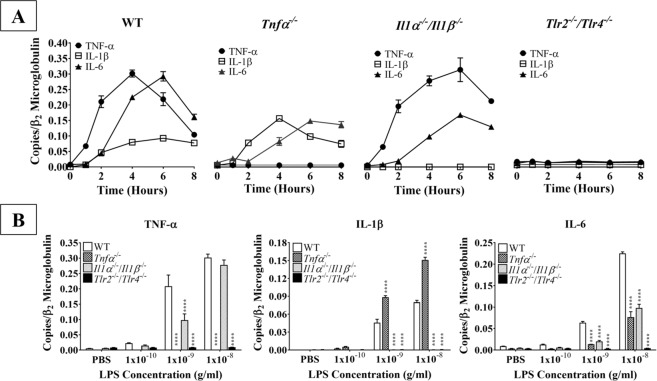
Kinetics of pro-inflammatory cytokine production by LPS-stimulated WT and knockout astrocytes. (**A**) Purified and subcultured murine WT, *Tnfα*^*−/−*^, *Il1α*^*−/−*^*/Il1β*^*−/−*^, and *Tlr2*^*−/−*^*/Tlr4*^*−/−*^ astrocytes were incubated with graded concentrations of freshly prepared bacterial LPS (0–10 ng/mL). Cultures were terminated at 0, 1, 2, 4, 6, or 8 h after stimulation. Total RNA was then extracted, and mRNA expression of TNF-α, IL-1β, and IL-6 was determined by qPCR (n = 3, results show one representative experiment out of 4). **(B)** Purified and subcultured murine WT, *Tnfα*^*−/−*^, *Il1α*^*−/−*^*/Il1β*^*−/−*^, and *Tlr2*^*−/−*^*/Tlr4*^*−/−*^ astrocytes were incubated with graded concentrations of freshly prepared bacterial LPS (0–10 ng/mL). Cultures were terminated 4 h after stimulation. Total RNA was then extracted, and mRNA expression of TNF-α, IL-1β, and IL-6 was determined by qPCR. *p < 0.05; **p < 0.01; ***p < 0.001; ****p < 0.0001 (n = 3, results show one representative experiment out of 4).

Based on our findings that bacterial LPS promotes astrocytes production of pro-inflammatory cytokines, we next determined how expression of these cytokines is regulated. To test this, we isolated total RNA and then mRNA from primary astrocytes obtained from WT, *Tnfα*^*−/−*^, *Il1α*^*−/−*^*/Il1β*^*−/−*^, and *Tlr2*^*−/−*^*/Tlr4*^*−/−*^ mice following stimulation with 10 ng/mL LPS for various lengths of time (0–8 h) or with graded concentrations of LPS (0.1 ng/mL – 10 ng/mL) for four hours. This revealed that WT astrocytes exhibited a temporal delay in the upregulation of IL-1β and IL-6 mRNA expression following LPS stimulation, as neither cytokine was significantly upregulated until two hours post-stimulation (Figs. 1 and 4A). In contrast, just one hour of LPS stimulation significantly upregulated TNF-α mRNA expression (Figs. 1 and 4A). Though the mRNA expression of TNF-α and IL-6 eventually reached comparable levels, TNF-α mRNA expression peaked two hours before that of IL-6 (at 4 h and 6 h, respectively), confirming that TNF-α production is upstream of IL-6. Alternatively, IL-1β mRNA expression remained relatively stable from two to eight hours of LPS stimulation.

Interestingly, knockout astrocytes demonstrated altered kinetics and maximal expression of these pro-inflammatory cytokines relative to WT astrocytes (Fig. 4). Specifically, IL-6 mRNA upregulation was delayed in both *Tnfα*^*−/−*^ and *Il1α*^*−/−*^*/Il1β*^*−/−*^ astrocytes, with no significant increase in production until four hours post-stimulation. Importantly, in these astrocytes, IL-6 mRNA production still peaked at six hours post-stimulation (0.14 and 0.16 copies/β_2_ microglobulin, respectively). However, the maximal amount of IL-6 mRNA produced by astrocytes from these two knockouts was half that seen in WT astrocytes (0.29 copies/β_2_ microglobulin at peak). In contrast, IL-1β mRNA expression clearly peaked at four hours post-stimulation in *Tnfα*^*−/−*^ astrocytes. In *Il1α*^*−/−*^*/Il1β*^*−/−*^ astrocytes, peak TNF-α mRNA expression was comparable to that seen in WT astrocytes (0.31 and 0.30 copies/β_2_ microglobulin, respectively). While WT astrocyte TNF-α mRNA peaked at four hours, TNF-α expression did not decline in *Il1α*^*−/−*^*/Il1β*^*−/−*^ astrocytes until after six hours following LPS stimulation. Therefore, our findings clearly demonstrated that TNF-α and IL-1β positively control IL-6 production. Finally, as expected, *Tnfα*^*−/−*^ and *Il1α*^*−/−*^*/Il1β*^*−/−*^ astrocytes did not produce mRNA for the respective cytokines, confirming their genotypes. Additionally, *Tlr2*^*−/−*^*/Tlr4*^*−/−*^ astrocytes did not produce any pro-inflammatory cytokines, demonstrating that in astrocytes, LPS acts exclusively through these two innate immune receptors and to activate astrocytes.

Next, to determine the mechanisms underlying the TLR2/4 signaling pathway, we further examined how different concentrations of LPS affected the mRNA expression of pro-inflammatory cytokines. To test this, we analyzed total RNA from astrocytes isolated from newborn WT, *Tnfα*^*−/−*^, *Il1α*^*−/−*^*/Il1β*^*−/−*^, and *Tlr2*^*−/−*^*/Tlr4*^*−/−*^ mice that were stimulated with graded concentrations of LPS (0.1–10 ng/mL) or vehicle control (PBS, pH 7.4) for four hours (Fig. 4B). In the vehicle control and low LPS (0.1 ng/mL) conditions, pro-inflammatory cytokine mRNA expression (TNF-α, IL-1β, IL-6) did not significantly differ between any of the genotypes. *Il1α*^*−/−*^*/Il1β*^*−/−*^ astrocytes produced significantly less TNF-α mRNA than WT astrocytes stimulated with 1 ng/mL LPS (*p* < 0.0001), but this difference was attenuated at the highest concentration of LPS (10 ng/mL). Interestingly, *Tnfα*^*−/−*^ astrocytes produced significantly more IL-1β mRNA than WT astrocytes (*p* < 0.0001) when stimulated with either 1 or 10 ng/mL LPS. IL-6 mRNA production was significantly lower in both *Tnfα*^*−/−*^ and *Il1α*^*−/−*^*/Il1β*^*−/−*^ astrocytes (*p* < 0.0001) in the 1 and 10 ng/mL LPS conditions. As expected, *Tlr2*^*−/−*^*/Tlr4*^*−/−*^ astrocytes expressed negligible amounts of TNF-α, IL-1β, and IL-6 mRNAs relative to WT astrocytes (*p* < 0.0001) in both the 1 and 10 ng/mL LPS conditions. Additionally, TNF-α and IL-1β mRNAs were not detected in *Tnfα*^*−/−*^ and *Il1α*^*−/−*^*/Il1β*^*−/−*^ astrocytes, respectively, in any of the conditions, confirming their genotype. Together, these results demonstrate that while TNF-α production is upstream of IL-1β and IL-6, these three pro-inflammatory cytokines modulate the expression of one another.

### Pro-inflammatory cytokines modulate the production of chemokines by LPS-stimulated astrocytes

**Figure 5:**
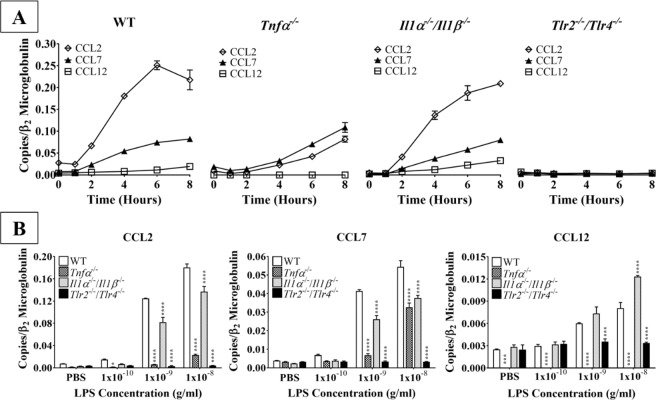
Kinetics of CCR2 ligand production by LPS-stimulated WT and knockout astrocytes. (**A**) Purified and subcultured murine WT, *Tnfα*^*−/−*^, *Il1α*^*−/−*^*/Il1β*^*−/−*^, and *Tlr2*^*−/−*^*/Tlr4*^*−/−*^ astrocytes were incubated with graded concentrations of freshly prepared bacterial LPS (0–10 ng/mL). Cultures were terminated at 0, 1, 2, 4, 6, or 8 h after stimulation. Total RNA was then extracted, and mRNA expression of CCL2, CCL7, and CCL12 was determined by qPCR. **(B)** Purified and subcultured murine WT, *Tnfα*^*−/−*^, *Il1α*^*−/−*^*/Il1β*^*−/−*^, and *Tlr2*^*−/−*^*/Tlr4*^*−/−*^ astrocytes were incubated with graded concentrations of freshly prepared bacterial LPS (0–10 ng/mL). Cultures were terminated 4 h after stimulation. Total RNA was then extracted, and mRNA expression of CCL2, CCL7, and CCL12 was determined by qPCR. *p < 0.05; **p < 0.01; ***p < 0.001; ****p < 0.0001 (n = 3, results show one representative experiment out of 4).

Next, we examined the mechanisms underlying LPS-induced CCR2 ligand expression in astrocytes (Fig. 5). As discussed above, in WT astrocytes, CCL2 was the CCR2 ligand most strongly induced by LPS (Fig. 2). The kinetics of CCL2 mRNA expression were similar to those of IL-6, with expression being significantly upregulated beginning at two hours and peaking at six hours post-stimulation (Figs. 2 and 5A). CCL7 mRNA expression also began to increase at two hours following LPS stimulation and gradually peaked at eight hours, though its expression was less than one-third that of CCL2. Finally, while CCL12 mRNA was significantly upregulated by LPS (Fig. 2), its expression was negligible compared to that of CCL2 or CCL7 (Fig. 5). Interestingly, CCL2 mRNA expression was markedly downregulated in *Tnfα*^*−/−*^ astrocytes, and its kinetics were different from those seen in WT astrocytes. While in WT astrocytes, CCL2 mRNA expression peaked at six hours post-LPS, the highest expression was observed at eight hours in *Tnfα*^*−/−*^ astrocytes. Alternatively, CCL2 mRNA expression was slightly lower in *Il1α*^*−/−*^*/Il1β*^*−/−*^ astrocytes compared to WT astrocytes at the earlier time points, but by eight hours following LPS stimulation, its expression was comparable between the two genotypes (Fig. 5A). This suggests that IL-1β has little, if any, feedback control of CCL2 mRNA expression. The kinetics of CCL7 mRNA expression were relatively unchanged in both LPS-stimulated *Tnfα*^*−/−*^ and *Il1α*^*−/−*^*/Il1β*^*−/−*^ astrocytes compared to WT astrocytes, suggesting a CCL2-independent regulatory mechanism. Alternatively, while LPS similarly upregulated CCL12 mRNA in both WT and *Il1α*^*−/−*^*/Il1β*^*−/−*^ astrocytes, CCL12 mRNA expression was negligible in *Tnfα*^*−/−*^ astrocytes. This demonstrates the dependence of CCL12 mRNA upregulation on TNF-α in LPS-stimulated astrocytes. Finally, as expected, LPS did not upregulate the expression of any CCR2 ligands in *Tlr2*^*−/−*^*/Tlr4*^*−/−*^ astrocytes, confirming again that LPS acts exclusively on TLR2/4.

We further examined how TNF-α and IL-1β modulated the mRNA expression of the CCR2 ligands CCL2, CCL7, and CCL12. To test this, we stimulated WT, *Tnfα*^*−/−*^, *Il1α*^*−/−*^*/Il1β*^*−/−*^, and *Tlr2*^*−/−*^*/Tlr4*^*−/−*^ astrocytes with graded concentrations of LPS for four hours, as described above (Fig. 5B). In the vehicle control condition, there was no significant difference in CCL2 or CCL7 mRNA production between WT astrocytes and any of the knockouts. Interestingly, at the lowest LPS concentration (0.1 ng/mL), CCL2 mRNA production was significantly lower in *Tnfα*^*−/−*^ astrocytes compared to WT (*p* < 0.05). CCL2 mRNA expression was also markedly downregulated in *Tnfα*^*−/−*^ astrocytes stimulated with higher concentrations of LPS compared to WT astrocytes (*p* < 0.001). CCL2 production by *Il1α*^*−/−*^*/Il1β*^*−/−*^ astrocytes was also significantly lower than that seen in WT astrocytes (*p* < 0.0001); however, this downregulation was not as profound as that seen in *Tnfα*^*−/−*^ astrocytes. Similarly, CCL7 mRNA was significantly downregulated in *Tnfα*^*−/−*^ and *Il1α*^*−/−*^*/Il1β*^*−/−*^ astrocytes compared to WT (*p* < 0.0001) treated with either 1 or 10 ng/mL LPS. Interestingly, LPS stimulation did not upregulate CCL12 mRNA in *Tnfα*^*−/−*^ astrocytes compared to WT astrocytes (*p* < 0.001) (Fig. 5B). This supports the notion that TNF-α regulates CCL12 mRNA expression in LPS-stimulated astrocytes. Conversely, CCL12 mRNA production was significantly upregulated in *Il1α*^*−/−*^*/Il1β*^*−/−*^ astrocytes stimulated with 10 ng/mL LPS compared to WT astrocytes. Finally, as expected, CCL2, CCL7, and CCL12 mRNA production following LPS stimulation in *Tlr2*^*−/−*^*/Tlr4*^*−/−*^ astrocytes was comparable to that of the vehicle control. Again, this demonstrates the necessity of TLR2/4 for LPS-induced astrocyte activation.

**Figure 6:**
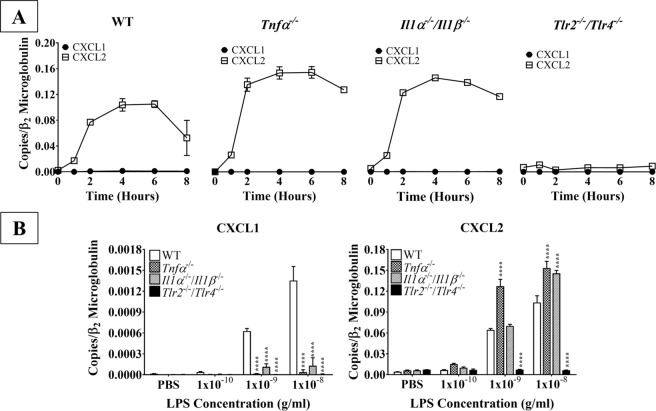
Kinetics of neutrophil chemokine production by LPS-stimulated WT and knockout astrocytes. (**A**) Purified and subcultured murine WT, *Tnfα*^*−/−*^, *Il1α*^*−/−*^*/Il1β*^*−/−*^, and *Tlr2*^*−/−*^*/Tlr4*^*−/−*^ astrocytes were incubated with graded concentrations of freshly prepared bacterial LPS (0–10 ng/mL). Cultures were terminated at 0, 1, 2, 4, 6, or 8 h after stimulation. Total RNA was then extracted, and mRNA expression of CXCL1 and CXCL2 was determined by qPCR. **(B)** Purified and subcultured murine WT, *Tnfα*^*−/−*^, *Il1α*^*−/−*^*/Il1β*^*−/−*^, and *Tlr2*^*−/−*^*/Tlr4*^*−/−*^ astrocytes were incubated with graded concentrations of freshly prepared bacterial LPS (0–10 ng/mL). Cultures were terminated 4 h after stimulation. Total RNA was then extracted, and mRNA expression of CXCL1 and CXCL2 was determined by qPCR. *p < 0.05; **p < 0.01; ***p < 0.001; ****p < 0.0001 (n = 3 results show one representative experiment out of 4).

Next, we examined how LPS stimulation altered the kinetics of neutrophil chemokine expression by stimulating WT, *Tnfα*^*−/−*^, *Il1α*^*−/−*^*/Il1β*^*−/−*^, and *Tlr2*^*−/−*^*/Tlr4*^*−/−*^ astrocytes with graded concentrations of LPS for four hours, as described above. In WT astrocytes, CXCL1 and CXCL2 mRNAs were both significantly upregulated by LPS in time- and concentration-dependent manners (Figs. 3 and 6). However, CXCL1 mRNA expression was negligible compared to that of CXCL2 in all LPS-stimulated astrocytes, regardless of genotype (Fig. 6). Interestingly, the kinetics of CXCL2 expression were similar between WT, *Tnfα*^*−/−*^, and *Il1α*^*−/−*^*/Il1β*^*−/−*^ astrocytes. Specifically, expression began to rise 1 hour following LPS stimulation, plateaued from 2 to 6 hours, and declined between 6 and 8 hours. However, the maximal CXCL2 mRNA expression in both *Tnfα*^*−/−*^ and *Il1α*^*−/−*^*/Il1β*^*−/−*^ astrocytes (0.15 and 0.14 copies/β_2_ microglobulin, respectively) was about 50% higher than that of WT astrocytes (0.10 copies/β_2_ microglobulin), suggesting that both TNF-α and IL-1β negatively regulate CXCL2 mRNA expression. Finally, LPS stimulation did not increase CXCL2 expression in *Tlr2*^*−/−*^*/Tlr4*^*−/−*^ astrocytes, as expected.

Finally, we further examined how TNF-α and IL-1β regulated CXCL1 and CXCL2 mRNA expression in LPS-stimulated astrocytes. As described above, WT, *Tnfα*^*−/−*^, *Il1α*^*−/−*^*/Il1β*^*−/−*^, and *Tlr2*^*−/−*^*/Tlr4*^*−/−*^ astrocytes were stimulated with graded concentrations of LPS for four hours and then CXCL1 and CXCL2 mRNA expression was measured via qPCR. This revealed that CXCL1 and CXCL2 mRNA production did not significantly differ between any of the knockouts and WT astrocytes in the vehicle control or low LPS (0.1 ng/mL) conditions (Fig. 6B). However, CXCL1 mRNA expression was significantly lower in all three knockouts as compared to WT astrocytes when treated with higher concentrations of LPS (1 ng/mL or 10 ng/mL; *p* < 0.0001). Alternatively, *Tnfα*^*−/−*^ astrocytes produced significantly more CXCL2 than WT astrocytes in the medium (1 ng/mL) and high (10 ng/mL) LPS conditions (*p* < 0.0001). *Il1α*^*−/−*^*/Il1β*^*−/−*^ astrocytes also produced significantly more CXCL2 mRNA as compared to WT astrocytes, but only when stimulated with 10 ng/mL LPS (*p* < 0.0001). As expected, *Tlr2*^*−/−*^*/Tlr4*^*−/−*^ astrocytes expressed negligible amounts of CXCL1 and CXCL2 mRNAs compared to WT astrocytes in both the medium (1 ng/mL) and high (10 ng/mL) LPS conditions, confirming the necessity of TLR2/4 for the effects of LPS stimulation. Taken together, these results suggest that both TNF-α and IL-1β critically modulate the expression of these two neutrophil-active chemokines, i.e., positively regulating CXCL1 and negatively regulating CXCL2.

### TNF-α acts as an autocrine/paracrine to regulate cell cycle progression in LPS-stimulated astrocytes

**Figure 7:**
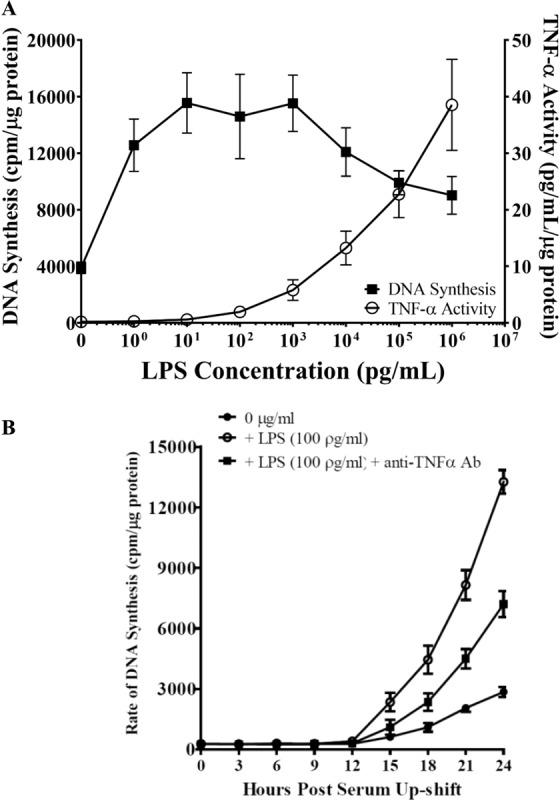
(**A**) Production of bioactive TNF-α by serum-deprived astrocytes as they re-enter cell cycle following LPS stimulation. Newborn rat astrocytes were rendered into G_0_ phase by serum deprivation and then allowed to re-enter the cell cycle via serum up-shift in the presence of graded concentrations of LPS. After a 24-h incubation, cell-free medium was collected to measure bioactive TNFα production and the rate of DNA synthesis using the WEHI and ^3^H-thymidine incorporation assays, respectively (n = 7–9 results show an average of three experiments). **(B)** LPS-stimulated cell cycle re-entry in astrocytes is TNF-α-dependent. Newborn astrocytes were rendered into G_0_ phase by serum deprivation and then allowed to re-enter the cell cycle via serum up-shift in the presence of freshly prepared LPS (100 pg/ml) with or without anti-TNF-α antibodies (10 U/mL). The ^3^H-thymidine incorporation assay was performed to measure rate of DNA synthesis. WT vs. LPS treatment: ***p < 0.05; **p < 0.001; *p < 0.0001. LPS treatment group vs. LPS + anti-TNF-α antibodies: ^!^p < 0.001; ^!!^p < 0.01; ^!!!^p < 0.05 (n = 7–9 results show an average of three experiments).

We next examined the mechanisms underlying TLR2/4-induced proliferation in LPS-stimulated astrocytes. Because the production of several pro-inflammatory cytokines and chemokines (i.e., CCL2) downstream of TLR2/4 activation was TNF-α-dependent (Figs. 4 and 5), it was conceivable that astrocyte proliferation also depended on TNF-α. Thus, we hypothesized that TNF-α functioned as an autocrine/paracrine of astrocyte effector functions following LPS stimulation. Without confining our findings in murine astrocytes, in the following studies, newborn rat astrocytes were used. In brief, rat astrocytes were subcultured in 6-well plates (10^4^ cells/cm^2^), grown to 30–50% confluence, and then rendered to the G_0_ phase by serum deprivation^23–25^. Following this 48-h serum deprivation period, astrocytes were overlaid with fresh medium (10% BCS/DMEM) to allow re-entry into the G_1_ phase in the presence of graded concentrations of LPS (1 pg/ml – 1 μg/ml) for 24 h. Cells were then pulse-labeled with ^3^H-thymidine for one hour to evaluate DNA synthesis, while cell-free conditioned medium was collected to measure TNF-α bioactivity with the WEHI cytolytic assay. Our results (Fig. 7A) showed that LPS promoted astrocyte proliferation in a concentration-dependent manner, with peak DNA synthesis occurring at LPS concentrations between 10 pg/ml – 1 ng/ml. Astrocyte production of bioactive TNFα in the presence of graded concentrations of LPS followed a completely different pattern such that TNFα levels continued to increase at the highest concentration of LPS tested (1 μg/ml), albeit also in a concentration-dependent manner.

We next determined whether the bioactive TNF-α released by LPS-stimulated astrocytes was essential to the increased astrocyte proliferation. To test this, serum-deprived rat astrocytes were stimulated with LPS (100 pg/ml) and with or without neutralizing polyclonal anti-TNF-α antibodies (10 U/mL). The extent of S-phase progression, as determined by DNA synthesis, was then quantified every three hours using the ^3^H-thymidine incorporation method. This revealed that LPS significantly upregulated ^3^H-thymidine incorporation during S phase (12–24 h post-serum up-shift) in both the LPS alone and LPS + anti-TNF-α antibodies groups compared to vehicle-treated astrocytes (Fig. 7B). However, anti-TNF-α antibodies significantly limited ^3^H-thymidine incorporation during S phase in the treatment group as compared to astrocytes treated with LPS alone. This suggests that TNF-α is an important autocrine/paracrine for astrocyte cell cycle progression following inflammatory stimulation. Furthermore, both LPS treatment and TNF-α inhibition did not alter the length of the G_1_ phase, as determined by the kinetics of ^3^H-thymidine incorporation.

## Discussion

Despite being the most abundant type of glia in the CNS, astrocytes are vastly understudied and their functions remain poorly defined. In particular, the innate immune functions of astrocytes have been largely ignored until recently due to a widespread belief that microglia are the sole CNS effector immune cells^26–29^. Importantly, it has been shown that astrocytes, like microglia, can become activated in pathological states such as infection, traumatic brain injury, and neurodegenerative diseases, including Alzheimer’s disease (AD), Parkinson’s disease (PD), and amyotrophic lateral sclerosis (ALS)^30^. This activation leads to astrocyte hypertrophy, proliferation, and production of pro-inflammatory mediators. However, the mechanisms underlying the transition from normal, quiescent astrocytes to reactive astrocytes are poorly understood. Given the mounting evidence that chronic inflammation contributes to numerous neurological diseases, the elucidation of these unknown pathways is imperative, both for unraveling their pathogenesis and for identifying potential therapeutic targets. Astrocytes have been shown to express TLRs^15^; however, their roles in astrocyte activation have not been fully explored. TLRs confer a wide variety of functions on different CNS cell subtypes, including classical innate immune functions in microglia^13,14^, cell fate determination in neural progenitor cells^9,10^, regulation of neurite outgrowth in developing neurons^11,12^, and cell death in oligodendrocyte precursor cells^8^. In this study, we systemically examined the role of TLR2/4 and their downstream effector functions and regulatory mechanisms in astrocytes.

Although TLR2 and TLR4 were shown to be expressed on astrocytes over a decade ago^31^, the data surrounding their functionality and contribution to neuroinflammation are controversial. Some studies suggest that astrocytes cannot be activated without microglial priming^32–34^; however, these studies were either performed on specific subpopulations of astrocytes or proposed that *in vitro* experiments on astrocytes were contaminated with microglia. Here, we showed that our astrocyte cultures contained less than 0.4% microglial contamination (Fig. S1), largely removing this concern. Additionally, our cultures were generated from the entire cerebral cortex, allowing for the examination of astrocyte innate immune functions on a global population level. Our results showed that the highly potent exogenous TLR2/4 agonist bacterial LPS significantly upregulated astrocyte mRNA expression of inflammatory mediators in time- and concentration-dependent manners, including the pro-inflammatory cytokines TNF-α, IL-1β, and IL-6 (Fig. 1), the CCR2 ligands CCL2, CCL7, and CCL12 (Fig. 2), and the neutrophil-active chemokines CXCL1 and CXCL2 (Fig. 3). Importantly, none of these pro-inflammatory mediators were upregulated in astrocytes derived from *Tlr2*^*−/−*^/*Tlr4*^*−/−*^ mice (Figs. 4–6), confirming that LPS activates astrocytes exclusively through TLR2 and TLR4. Ultimately, our findings demonstrate that exogenous pathogenic ligands can promote classical immune functions in astrocytes via TLR2/4. These data add to a growing body of evidence supporting that astrocytic PRRs contribute directly to neuroinflammation^35,36^. Our findings also show that astrocytes play an important role in non-sterile inflammation and are likely significant players in host immune responses.

Interestingly, LPS-stimulated astrocytes differentially expressed various pro-inflammatory cytokines and chemokines. Particularly, LPS-stimulated WT astrocytes strongly upregulated TNF-α (Figs. 1 and 4), and TNF-α was the first pro-inflammatory cytokine upregulated by LPS-stimulated astrocytes (Fig. 4). Other studies have shown that high TNF-α levels correlate with cognitive impairment in different animal models, including the experimental autoimmune encephalitis model of multiple sclerosis^37^, postoperative cognitive decline^38^, and prion disease^39^. TNF-α is also elevated in several human neurodegenerative conditions such as AD^40–43^, PD^44^, and HIV-associated dementia^45^. Taken together, these findings and our results showing TNF-α production by TLR2/4-activated astrocytes strongly suggest that activated astrocytes and their downstream effector TNF-α play critical roles in innate immunity and CNS pathogenesis.

We also showed that CCL2 was the most abundantly produced CCR2 ligand in LPS-stimulated astrocytes. Specifically, these astrocytes produced 3 times as much CCL2 as CCL7, and 22 times as much CCL2 as CCL12. Importantly, CCL2 is responsible for recruiting peripheral monocytes in response to viral infection^46,47^. Additionally, resident microglia and peripheral monocytes are recruited and activated via CCR2 in the APPSwe model of AD-like disease^16^. Elevated levels of CCL2 have also been demonstrated in cases of bacterial meningitis^48^ and in AD patients^49,50^. Thus, our results show that astrocytes may recruit microglia and monocytes to areas of injury (i.e., infectious agents, damage-associated molecular patterns) by producing CCR2-active chemokines, especially CCL2.

**Figure 8:**
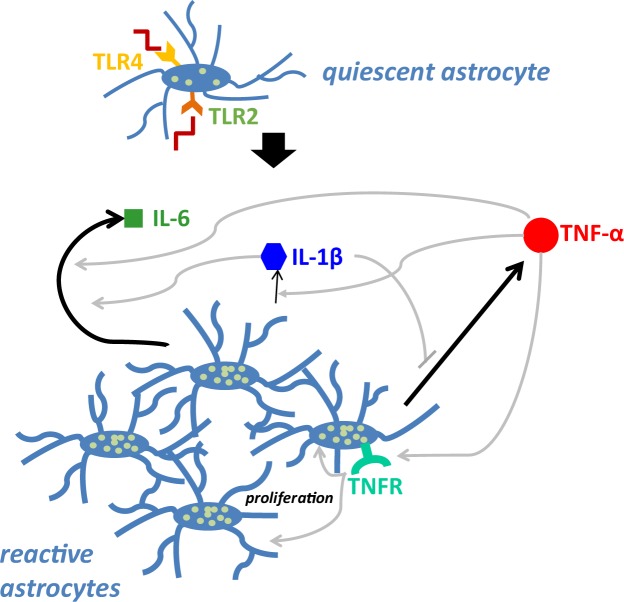
The self-regulatory mechanisms underlying pro-inflammatory cytokine production following TLR2/4 activation. Feedback pathways were determined by quantifying mRNA production of each pro-inflammatory cytokine following stimulation with bacterial LPS. Arrow thickness indicates the relative magnitude of peak production. Black arrows indicate direct production of mediators by astrocytes; gray arrows indicate mechanisms of self-regulation, as determined by the effect of various knockouts on cytokine expression. Arrows with pointed heads indicate positive regulation, while arrows with flat, perpendicular lines indicate negative regulation. White dots on astrocytes represent GFAP expression.

In addition to demonstrating the functional significance of astrocytic TLR2 and TLR4, we also found that the downstream effectors act in a ligand-specific self-regulatory manner (Fig. 8). Specifically, in response to TLR2/4 activation, TNF-α negatively regulated IL-1β expression, as TLR2/4 stimulation by LPS strongly upregulated IL-1β expression in *Tnfα*^*−/−*^ astrocytes (Fig. 4). Alternatively, LPS stimulation in *Il1α*^*−/−*^*/Il1β*^*−/−*^ astrocytes significantly downregulated TNF-α expression, suggesting that IL-1β acts as a positive regulator of TNF-α production in response to TLR2/4 activation (Fig. 4). Furthermore, TNF-α and IL-1β both positively regulated IL-6 production, as IL-6 expression was significantly lower in LPS-stimulated *Tnfα*^*−/−*^ and *Il1α*^*−/−*^*/Il1β*^*−/−*^ astrocytes (Fig. 4). Our findings of the self-regulatory mechanisms underlying pro-inflammatory cytokine production are summarized in Fig. 8.

We found similar results regarding the expression of various CCR2 ligands. In response to TLR2/4 activation, TNF-α strongly induced CCL2 production, as shown by the markedly decreased CCL2 production by LPS-stimulated *Tnfα*^*−/−*^ astrocytes (Fig. 5). LPS-induced IL-1β also induced CCL2 expression, as shown by the significant decrease in CCL2 production by LPS-stimulated *Il1α*^*−/−*^*/Il1β*^*−/−*^ astrocytes; however, this induction was weaker than that of TNF-α (Fig. 5). Following TLR2/4 activation, both TNF-α and IL-1β positively regulated CCL7 production, as shown by the significant downregulation of CCL7 expression in LPS-stimulated *Tnfα*^*−/−*^ and *Il1α*^*−/−*^*/Il1β*^*−/−*^ astrocytes (Fig. 5). Finally, both TNF-α and IL-1β positively regulated CCL12 in response to TLR2/4 stimulation, as shown by the significant decrease in CCL12 expression in both *Tnfα*^*−/−*^ and *Il1α*^*−/−*^*/Il1β*^*−/−*^ astrocytes relative to WT astrocytes (Fig. 5). Thus, CCL2 likely plays a major role in recruiting microglia and monocytes, given the markedly higher levels of CCL2 than those of CCL7 and CCL12 produced by LPS-stimulated astrocytes; future recruitment essays will be able to confirm such assertion. Furthermore, our findings show that following TLR2/4 activation in LPS-stimulated astrocytes, TNF-α serves as a predominant “proxy” for CCL2 upregulation.

TNF-α and IL-1β also modulated expression of neutrophil-active chemokines. Specifically, they both positively regulated CXCL1 expression following TLR2/4 activation, as shown by the significant decrease in CXCL1 expression in both *Tnfα*^*−/−*^ and *Il1α*^*−/−*^*/Il1β*^*−/−*^ astrocytes compared to WT astrocytes stimulated with either ligand (Fig. 6). Conversely, TNF-α and IL-1β both negatively regulated the production of CXCL2 downstream of TLR2/4, as shown by the significant decrease in CXCL2 production in *Tnfα*^*−/−*^ and *Il1α*^*−/−*^*/Il1β*^*−/−*^ astrocytes compared to LPS-stimulated WT astrocytes (Fig. 6). CXCL2 is likely the predominant neutrophil-active chemokine produced following TLR2/4 stimulation, as evidenced by the markedly higher levels of CXCL2 than CXCL1.

Finally, as discussed above, cell cycle re-entry and active proliferation are part of the switch from quiescent to reactive astrocytes; however, the mechanisms underlying this transition are largely unknown. Here, we showed that LPS-induced activation of TLR2/4 enhanced bioactive TNF-α production (Fig. 7A) and astrocyte proliferation (Fig. 7A,B). We further showed that TNF-α positively regulated astrocyte proliferation downstream of TLR2/4 in an autocrine/paracrine manner, as addition of neutralizing anti-TNF-α antibodies significantly lowered LPS-induced proliferation in primary rodent astrocytes (Fig. 7B). While other studies have demonstrated the functional role of IL-1 and signaling pathway of TLR4 in astrocytes^51,52^, to our knowledge, this is the first time the critical autocrine/paracrine effects of TNF-α on astrocyte functioning are demonstrated. In addition, other studies have demonstrated the roles of TLRs, including TLR2 and TLR4, in upregulating proliferation in various cancers^53–56^, to our knowledge, we are the first to demonstrate their significance in the transition between quiescent and proliferative astrocytes via the production of TNF- α. Our findings will likely delineate the cellular and molecular mechanisms involved in different neurological conditions.

Our findings demonstrate that TLR2/4 signaling in astrocytes promotes classical innate immune functions, including increased mRNA expression of pro-inflammatory cytokines and chemokines. Furthermore, the pro-inflammatory cytokines TNF-α, IL-β, and IL-6 act in a self-regulatory manner. While both TNF-α and IL-1β modulate the downstream expression of CCR2 chemokines (CCL2, CCL7, CCL12) and neutrophil-active chemokines (CXCL1, CXCL2), TNF-α is the predominant regulator of these chemokines in LPS-stimulated astrocytes. TLR2/4 signaling also promotes astrocyte cell cycle re-entry and proliferation in a TNF-α-dependent manner. These findings have important implications for the role of astrocytes in the pathogenesis of several neurological diseases involving innate immunity and inflammation, including infections, autoimmune diseases (i.e., multiple sclerosis), traumatic brain injury, and neurodegenerative diseases^30^. Thus, future research on both the pathogenesis of these diseases and their potential therapeutic interventions should consider the contribution of astrocytes.

## Materials and Methods

### Mice

All experimental procedures were approved by the Institutional Animal Care and Use Committee (IACUC) at the Geisel School of Medicine at Dartmouth, and performed in accordance with approved protocols. Mice were purchased either from the National Cancer Institute (NCI) Mouse Repository (wild-type [WT]: C57BL/6NCr, Strain # 01C55) or Jackson Laboratories (*Tlr4*^*−/−*^; *Tlr2*^*−/−*^; *Tnfα*^*−/−*^). *Il1α*^*−/−*^/*Il1β*^*−/−*^ mice, which are well characterized and published in literature^57,58^, were a gift from Dr. Yoichiro Iwakura (Tokyo University of Science). Our novel *Tlr2*^*−/−*^/*Tlr4*^*−/−*^ mice were generated by cross-breeding *Tlr2*^*−/−*^ with *Tlr4*^*−/−*^ mice. All genotypes were confirmed by polymerase chain reaction (PCR) and gel electrophoresis. All colonies were bred and maintained in barrier facilities at the Center for Comparative Medicine and Research at Dartmouth College, with 12 h light/dark cycles (7 am to 7 pm) and ad libitum access to standard chow and water.

*Tlr2*^*−/−*^*/Tlr4*^*−/−*^ knockout mice were created by traditional breeding methods. Briefly, single knockout *Tlr2*^*−/−*^ mice were crossed with single knockout *Tlr4*^*−/−*^ mice to generate double heterozygous mice (*Tlr2*^*+/−*^*/Tlr4*^*+/−*^). F1 heterogenous mice were then inbred, and all their offspring (F2) were genotyped by PCR and gel electrophoresis. F2 mice containing two knockout alleles for both genes (*Tlr2*^*−/−*^*/Tlr4*^*−/−*^) were selected to establish the breeding colony. *Tlr2*^*−/−*^*/Tlr4*^*−/−*^ offspring were inbred for five generations and then genotyped by PCR to confirmation their genotype prior to being harvested for neonatal astrocytes. The *Tlr2*^*−/−*^*/Tlr4*^*−/−*^ colony also underwent random genotyping by PCR for gene confirmation every few generations.

### Rats

To eliminate species-specific outcomes, newborn Sprague Dawley rat astrocytes were also used for astrocyte activation and proliferation experiments, which did not require the use of knockouts (Fig. 7A,B). All experimental procedures were approved by the IACUC at The State University of New York (SUNY) at Buffalo and performed in accordance with the approved protocols. Newborn pups were purchased from Harlan Sprague Dawley and housed briefly in the Animal Research Center at The SUNY at Buffalo.

### Primary astrocyte culture

Newborn rodent pups (<48 h old) were first decapitated and then their brains were removed and placed in a cold 1:1 solution of phosphate-buffered saline (PBS, pH 7.4: Corning Cellgro, Manassas, VA; Catalog # 21-040-CV) and HyClone Ham’s Nutrient Mixture F12 (GE Healthcare Life Sciences, Logan, UT; Catalog # SH30025.01). Next, the cerebral cortices were isolated by microdissection, the meninges and blood vessels were removed, and the cortices were mechanically dissociated. The cell suspension was then filtered through 70-µm nylon mesh to remove tissue debris, centrifuged (800 × *g*, 5 min), and resuspended in growth media containing 10% fetal bovine serum (FBS: HyClone, Logan, UT) and 1% penicillin-streptomycin (v/v; Sigma, St. Louis, MO, Catalog # P4333) in Dulbecco’s modified Eagle’s medium (DMEM: Gibco/LifeTechnologies, Inc., Grand Island, NY). Cells were then plated at a density of one brain per flask in vent cap T75 flasks (Corning Inc., Corning, NY; Catalog # 3276) and maintained at 37 °C in a CO_2_ incubator (5% CO_2_/95% humidified air) until *in vitro* studies. A previous study showed that the initial astrocyte cultures generated by this method are ≥ 95% pure^23–25^, whereas our studies demonstrated pre-column purity of > 99% (Fig. S1, see below)

### Astrocyte purification

At the start of each experiment, astrocytes were further purified by magnetic microbead-based negative selection to remove microglia using the CD11b MicroBead kit (Miltenyi Biotec, Auburn, CA; Catalog # 130-093-634). After removing the medium, cells were briefly washed with cold Tris-EDTA (pH 7.4, Sigma Aldrich, Saint Louis, MO; Catalog # 93302) and then enzymatically detached from T75 flasks using cold 0.25% Trypsin-EDTA (Mediatech, Inc., Manassas, VA; Catalog # 25-053-CI). Next, the cells were pelleted by centrifugation (300 × *g*, 5 min) and resuspended in MACS buffer (0.5% w/v bovine serum albumin [BSA] in PBS). Cells were manually counted using a hemocytometer and then washed and incubated with CD11b MicroBeads according to the manufacturer’s protocol. Cell separation was performed using LS Columns (Miltenyi Biotec; Catalog # 130-042-401) and a MidiMACS Separator (Miltenyi Biotec; Catalog # 130-042-302) outfitted with 30-µm pre-separation filters (Miltenyi Biotec; Catalog # 130-041-407).

### Flow cytometric analysis of astrocyte purity

Astrocyte purity was determined by flow cytometric analysis for CD11b + cells. Briefly, the cells (both pre-purification and post-purification fractions) were washed with 1 mL of cold FACS buffer (1% BSA, 0.02% sodium azide in PBS) and then centrifuged (800 × *g*, 5 min) at room temperature. After aspirating the supernatant, cells were resuspended in 100 µL of blocking cocktail (98 µL of FACS buffer + 2 µL of rat anti-mouse CD16/CD32 [BD Biosciences, San Jose, CA; Catalog # 553141]) and incubated on ice for 20 min. Next, the cells were incubated with 5 μL of rat anti-mouse CD11b-APC (1:100 BSA/PBS; Millipore, Burlington, MA; Catalog # MABF520) and covered on ice for 30 min. Finally, the cells were washed twice with FACS buffer, resuspended in 200 μL of PBS, and analyzed using an 8-color MACSQuant-10 (Miltenyi Biotec) in the DartLab Flow Cytometry core at the Geisel School of Medicine. Although only 0.54% of “unpurified” cells were CD11b + , column purification lowered this percentage to 0.4% (Supplemental Fig. 1 [Fig. S1]).

### Astrocyte subculture and *in vitro* stimulation

#### Non-serum deprivation experiments

Purified primary murine astrocytes were seeded onto 12-well plates at a concentration of 1 × 10^4^ cells/cm^2^ in 10% bovine calf serum (BCS; HyClone, Logan, UT)/DMEM (v/v). After an initial 24-h incubation at 37 °C in 5% CO_2_/95% humidified air, the medium was removed and the cultures were washed once with PBS (pH 7.4) to remove cell debris and non-adherent cells. Next, 10% BCS/DMEM was added to adherent cells (2 mL), and the astrocytes were allowed to proliferate at 37 °C in 5% CO_2_/95% humidified air for 48 h. Following this incubation, the supernatant was replaced with fresh 10% BCS/DMEM (2 mL in 12-well plates, 3 mL in 6-well plates) in the presence of varying concentrations of LPS from *Escherichia coli K12* (LPS-EK: InvivoGen, San Diego, CA) dissolved in PBS (pH 7.4; PBS alone as a negative control). This step represented the start of the experiment (i.e., T_0_).

#### Serum deprivation experiments

Rat astrocytes were seeded onto 6-well plates at a concentration of 1 × 10^4^ cells/cm^2^ in 10% BCS/DMEM and then grown to 30–50% confluence at 37 °C in 5% CO_2_/95% humidified air. Following this incubation, the medium was removed, and the cells were rinsed with PBS (pH 7.4). The cells were then overlaid with 0.1% BCS/DMEM (3 mL) and incubated at 37 °C in 5% CO_2_/95% humidified air for 48 h such that by the end of the incubation period, 85–90% of the cells had entered cell cycle arrest^23–25^. At the end of serum deprivation, the medium was replaced with 10% BCS/DMEM (3 mL). This serum up-shift allowed the astrocytes to re-enter the cell cycle in a first-order manner. This step represented the start of the cell cycle entry experiment (i.e., T_0_)^23–25,59,60^. At T_0_, the cells were treated with freshly prepared LPS (in PBS, pH 7.4; final concentration: 100 pg/mL) with or without polyclonal anti-TNF-α antibodies (10 U/mL); PBS alone was used as a negative control.

#### ^3^H-thymidine incorporation assay

The incorporation of tritiated [methyl-^3^H]-thymidine into primary astrocytes was conducted according to established procedures to quantify cell proliferation^23–25,59,61^. Radio-labeled [methyl-^3^H]-thymidine (25 Ci/mmol; Amersham, Arlington Heights, IL) was added to each well 1 h prior to the termination of the experiment at a final activity of 1.0 μCi/ml (37 °C, 5% CO_2_/95% humidified air). At the end of the incubation, cultures were washed twice with 2 mL of Tris-EDTA buffer (pH 7.4) to remove excess ^3^H-thymidine. Then, DNA and total cellular proteins were extracted using the trichloroacetic acid (TCA) precipitation method^59,61^. Cell proliferation was measured as the incorporation of radioactivity per μg of protein in the acid-precipitated portion (cpm/μg protein). Tritium was quantified with a scintillation counter (LKB Wallac, Gaithersburg, MD) for beta particles for 10 min using an Ecoscint-A liquid scintillation cocktail (National Diagnostics, Manville, NJ). Total cellular protein was determined by the Bradford assay (Bio-Rad, Hercules, CA) using a microplate reader (model 3550-UV, Bio-Rad) at a wavelength of 595 nm.

### Trichloroacetic acid precipitation method

At the termination of these experiments, cells were washed twice with TEN buffer (40 mM Tris-HCl, pH 8.0; 1 mM EDTA; 150 mM NaCl) to remove excess ^3^H-thymidine and then frozen overnight (−20 °C) in TEN buffer to promote cell lysis. Upon thawing, the plates were mechanically scraped, and the lysates were transferred to 10-mL glass centrifuge tubes. TCA (Sigma Aldrich) was added to the lysates at a final concentration of 10% TCA (w/v) and then incubated overnight at 4 °C. The following day, lysates were centrifuged (800 × *g*, 20 min) at room temperature, and the supernatant was removed by aspiration. Next, 5% TCA was added (1 mL), and the precipitate was vortexed before being centrifuged again (800 × *g*, 20 min) at room temperature. The supernatant was aspirated again, 0.5 KOH (1 mL) was added, and the samples were incubated for 1 h at 4 °C. Tritium incorporation into DNA was determined as described above, and the protein concentration was determined using the Bradford assay (Bio-Rad, Hercules, CA).

### Total RNA isolation

At the end of each time point, the medium was removed from the 12-well plates, the cells were washed with warm PBS (pH 7.4), and adherent astrocytes were lysed using RLT buffer (Qiagen, Gaithersburg, MD) containing 0.55 mM 2-mercaptoethanol (Gibco/Life Technologies, Carlsbad, CA; Catalog # 21985023). Lysates were then flash frozen at −80 °C to further aid cell lysis. Plates were subsequently thawed on ice, and each well was gently scraped with a cell scraper (Celltreat, Pepperell, MA). Next, the total RNA was isolated from each well using the RNeasy Mini Kit (Qiagen; Catalog # 74106) and then quantified using a NanoDrop UV/Vis Spectrophotometer (ThermoFisher Scientific, Waltham, MA; Catalog # ND-2000).

### qPCR Analysis for mRNA expression

A total of 10–50 ng total RNA was used as template for the reverse transcriptase reaction. 50 μL cDNA was synthesized from each sample using the iScript cDNA synthesis kit (Bio-Rad, Hercules, CA; Catalog # 1708891). Reverse transcription was performed (1: 25 °C for 5 min; 2: 42 °C for 30 min; 3: 85 °C for 5 min) using a PTC-100 Peltier Thermal Cycler (MJ Research, Waltham, MA). Next, the real-time quantitative PCR (qPCR) reaction was performed using 2.5 µL of cDNA, 12.5 µL of iQ SYBR Green Supermix (Bio-Rad; Catalog # 1708884), and 400 nM sense and antisense primers. All oligonucleotide primers for qPCR (Supplemental Table 1 [Table S1]) were designed using the NCBI Primer-BLAST Design Tool and purchased from Integrated DNA Technologies (Coralville, IA). qPCR was performed using the CFX96 Real-Time System C1000 Touch Thermal Cycler (1: 95 °C for 3 min; 2: 95 °C for 15 s; 3: 55 °C for 15 s; 4: 72 °C for 30 s; 5: Repeat steps 2–4 39 times). Emitted fluorescence for each reaction was measured with a melt curve of 55–99 °C, with readings taken every 1 °C and held for 10 s. Amplification plots were analyzed using Bio-Rad CFX Manager software version 3.1. Gene expression was quantified by comparing the fluorescence generated by each sample with standard curves of known quantities, and the calculated number of copies was divided by the number of copies of the housekeeping gene β2 microglobulin.

### TNF-α Bioactivities

TNF-α bioactivities were measured in cell-free conditioned medium from astrocytic monolayers using WEHI 164 subclone 13 fibrosarcoma cells from mouse (ATCC, Manassas, VA, product number: CRL 1751) according to established procedures^62,63^. WEHI 164 fibrosarcoma cells are sensitive to the cytolytic effects of TNF-α. Cell-free supernatant (100 µl) from experimental astrocytic cultures was incubated with 100 µl of the WEHI cell suspension (5 × 10^5^ cells/mL) in 96-well plates at 37 °C in 5% CO_2_ humidified air for 20 h. Following this incubation, 180 µl of the supernatant were replaced by 180 µl of fresh culture medium. WEHI cells were then incubated with MTT (3[4,5-Dimethylthiazol-2-yl]-2,5-diphenyltetrazolium bromide; Thiazolyl blue) (Sigma Aldrich) for an additional 4 h. To solubilize the converted dye, 150 µl of the supernatant were replaced by 100 µl of 0.1 N HCl in absolute isopropanol. Plates were stored in darkness for 20 h. Next, the level of cell lysis was determined using a Bio-Rad Microplate Reader, model 3550-UV (Bio-Rad) at a wavelength of 540 nm. Units of bioactivity were calculated using internal recombinant TNF-α (rTNF-α) standards (Genzyme Diagnostics, Cambridge, MA) and expressed as pg/ml/µg protein.

### Statistical analysis

All experiments were performed at least three times, and all conditions were conducted in triplicate. The data were expressed as the mean ± standard error of the mean (SEM) and analyzed using Student’s t-tests for unpaired comparisons or Dunnett’s one-way analysis of variance (ANOVA) for multi-group comparisons, as appropriate. Analysis was performed using GraphPad Prism software. Statistical significance was defined as *p* < 0.05.

## Supplementary information

Supplementary figure and table.
