# Supplementary material for: Innate Immune Functions of Astrocytes are Dependent Upon Tumor Necrosis Factor-Alpha
Source: Sci Rep. 2020 Apr 27;10:7047. doi: 10.1038/s41598-020-63766-2 (PMC7184618; doi:10.1038/s41598-020-63766-2)
Supplement: Supplementary file 1 — Supplementary figure and table. [file 41598_2020_63766_MOESM1_ESM.pdf]

Innate Immune Functions of Astrocytes are Dependent Upon Tumor Necrosis Factor-Alpha

Kyla R. Rodgers[1], Yufan Lin[1], Thomas J. Langan[2,3], Yoichiro Iwakura[4], and Richard C. Chou[1]

[1]Department of Medicine, Geisel School of Medicine at Dartmouth, Dartmouth College, One Medical Center Drive, Lebanon, NH 03756, USA

[2]Departments of Neurology, Pediatrics, and Physiology and Biophysics, Jacobs School of Medicine and Biomedical Sciences, State University of New York at Buffalo, Buffalo, NY 14203, USA

[3]Hunter James Kelly Research Institute, New York State Center of Excellence Bioinformatics & Life Sciences, Buffalo, NY 14203, USA

[4]Research Institute for Biomedical Sciences, Tokyo University of Science, 2669 Yamazaki, Chiba 278-0022, Japan

CORRESPONDING ADDRESS:

Richard C. Chou, MD PhD
Department of Medicine
School of Medicine and Biomedical Sciences
University at Buffalo, State University of New York
Clinical and Translational Research Center, 6/F
875 Ellicott Street
Buffalo, NY 14203, USA
Email: richou@buffalo.edu

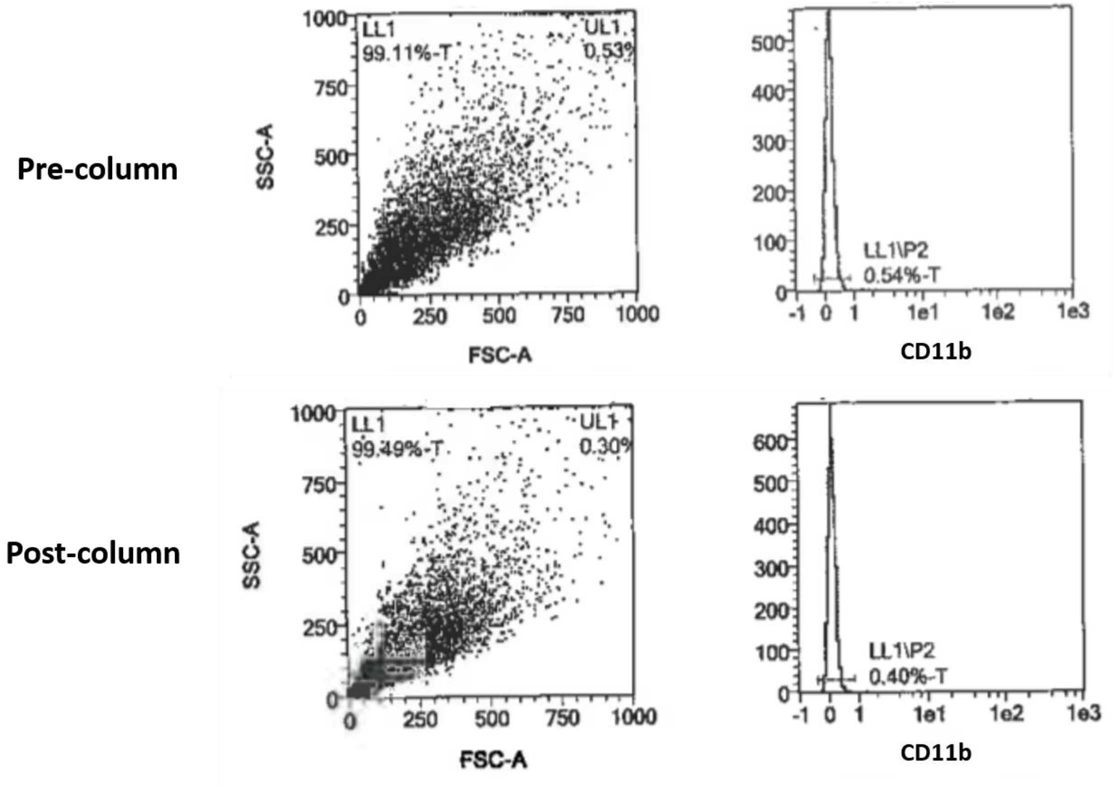

Figure S1: Percentage of CD11b+ cells in the culture both pre- and post-column purification. Confluent WT astrocyte cultures were trypsinized, resuspended in MACS buffer (0.5% w/v BSA in PBS). A small fraction of the cells were retained on ice without further treatment (pre-column). The remainder were manually counted, washed, and incubated with CD11b MicroBeads prior to negative selection of CD11b cells was the Miltenyi MACS magnetic bead-based purification (post-column). Following this purification, both fractions were re-suspended in FACS buffer, blocked with CD16/CD32, and stained for CD11b expression. Both groups were analyzed for CD11b expression using the 8-color MACSQuant10 (DartLab, Geisel School of Medicine at Dartmouth).

Target mRNA	Forward Primer Sequence	Reverse Primer Sequence	Accession number
**B2M**	CCGAACATACTGAACTGCTACGTAA	CCCGTTCTTCAGCATTTGGA	NM_009735.3
**TNF-α**	GCACAGAAAGCATGACCCG	GCCCCCCATCTTTTGGG	NM_013693
**IL-1β**	ACCTGTCCTGTGTAATGAAAGACG	TGGGTATTGCTTGGGATCCA	NM_008361
**IL-6**	TGTGGACATTCCTCACTGTGGTCA	ACATTCCAAGAAACCATCTGGCTAGGT	NM_031168.1
**CCL2**	CGGCTGGAGCATCCACGTGT	CTTTGGGACACCTGCTGCTGGT	NM_011333.3
**CCL7**	GTGTGGGCCCAACCAGATGGG	CAGCTTCCCAGGGACACCGAC	NM_013654.3
**CCL12**	GGCTGGACCAGATGCGGTGAG	CCGTGGGGAACTTCAGGGGGAA	NM_011331.2
**CXCL1**	GTGTTGCCCTCAGGGCC	GCCTCGCGACCATTCTTG	NM_008176.3
**CXCL2**	ACGCCCCCAGGACCC	CTTTTTGACCGCCCTTGAGA	NM_009140.2

Supplementary Table 1. qPCR primer sequences of all proinflammatory cytokines and chemokines used in the studies. B-microglobulin was used as housekeeping gene.
